# Draft Genome Sequence of Lactobacillus rhamnosus Strain CBC-LR1, Isolated from Homemade Dairy Foods in Bulgaria

**DOI:** 10.1128/MRA.00961-20

**Published:** 2020-09-17

**Authors:** Hanan R. Shehata, Richmond A. Chandler, Steven G. Newmaster

**Affiliations:** a NHP Research Alliance, College of Biological Sciences, University of Guelph, Guelph, Ontario, Canada; b Department of Microbiology, Faculty of Pharmacy, Mansoura University, Mansoura, Egypt; c Chandler Biopharmaceutical Corp., Milton, Ontario, Canada; University of Maryland School of Medicine

## Abstract

Here, we report the draft genome sequence of Lactobacillus rhamnosus strain CBC-LR1, which was isolated from naturally processed, homemade dairy foods in Bulgaria. The genome was assembled in 29 contigs with a total length of 2,892,155 bp and a GC content of 46.7%. Genome annotation predicted 2,638 coding genes and 49 tRNA genes.

## ANNOUNCEMENT

Lactobacillus rhamnosus is a species of lactic acid bacteria that is considered the most studied probiotic species for human use (1). This species can be found in fermented foods and in the intestinal and vaginal tracts, and strains belonging to this species have been reported to have human health benefits. For example, strain L. rhamnosus GG, the most widely studied probiotic strain, was found to have antimicrobial activity against *Salmonella enterica* serovar Typhimurium (2), Shigella sonnei (3), *Clostridium* spp., *Pseudomonas* spp., *Staphylococcus* spp., and *Streptococcus* spp. (4), as well as a role in controlling antibiotic-associated diarrhea (5, 6) and a role in alleviating nasal blockage in allergic rhinitis (7). Other L. rhamnosus strains were found to have antibacterial and antifungal activities in the urogenital tract (8) and anti‐inflammatory effects in patients with inflammatory bowel disease (9).

Lactobacillus rhamnosus strain CBC-LR1 was isolated from naturally processed, homemade dairy foods from an ecologically pure geographical area in Bulgaria in August 2001. L. rhamnosus strain CBC-LR1 was isolated on MRS agar plates that were incubated anaerobically at 37°C for 48 h.

Here, we report the genome sequence of Lactobacillus rhamnosus strain CBC-LR1. The genome was sequenced to obtain better insight into the probiotic properties and probiotic mechanisms of this strain and to evaluate its safety for human use.

Chandler Biopharmaceutical Corp. provided strain CBC-LR1 as a lyophilized powder. The NucleoSpin food kit (product number 740945.50; Macherey-Nagel, Germany) was used for genomic DNA extraction from 50 mg of the lyophilized powder, following the manufacturer’s instructions. A Qubit v4.0 fluorometer was used to quantify the extracted DNA using a Qubit double-stranded DNA (dsDNA) high-sensitivity (HS) assay kit. Genomic DNA was submitted to the Genomics Facility at the University of Guelph (Guelph, ON, Canada) for library preparation and sequencing on an Illumina MiSeq system using an Illumina Nextera XT kit and Illumina MiSeq reagent v3 kit (600 cycles; 2 × 300-bp reads).

CLC Genomics Workbench v20.0.3 (Qiagen Bioinformatics) was used to analyze the sequencing data. Default parameters were used except where otherwise noted. A quality-trimming step was performed to remove low-quality sequences (limit of 0.05 base-calling error probability) and to allow a maximum of 2 ambiguous nucleotides. The total numbers of paired reads before and after quality trimming were 1,684,162 and 1,683,896, respectively. High-quality reads with a total of 457,109,690 bases were used for *de novo* assembly, yielding ∼150× coverage. The genome was assembled in 29 contigs, with a total length of 2,892,155 bp, a GC content of 46.7%, and an *N*_50_ value of 179,274 bp. The full-length 16S rRNA gene sequence was extracted from the genome sequence using the ContEst16S tool (10) and was used for a BLAST search with GenBank to confirm species identity (11). The NCBI Prokaryotic Genome Annotation Pipeline (PGAP) v4.11 (https://www.ncbi.nlm.nih.gov/genome/annotation_prok) was used for genome annotation (12), which predicted 2,638 coding genes, 49 tRNA genes, and 6 rRNA genes.

### Data availability.

This whole-genome shotgun project has been deposited in DDBJ/ENA/GenBank under the accession number JACKWP000000000. The version described in this paper is version JACKWP000000000.1. The raw files were deposited in the SRA database under the accession number SRR12412204.
